# Examiner stratification reveals clinically relevant variability in large language model answers to endodontic patient questions

**DOI:** 10.3389/fmed.2026.1819087

**Published:** 2026-04-22

**Authors:** Saeed S. Alqahtani, Hmoud Ali Algarni, Meshal Aber Alonazi, Azhar Iqbal, Osama Khattak, Ravi Jothish, Mohmed Isaqali Karobari

**Affiliations:** 1 Department of Restorative Dental Sciences, College of Dentistry, Jouf University, Sakaka, Saudi Arabia; 2 Department of Conservative Dentistry and Endodontics, Saveetha Dental College and Hospitals, Saveetha Institute of Medical and Technical, Sciences, Saveetha University, Chennai, India

**Keywords:** artificial intelligence, communication, decision support systems, dentistry, endodontics, natural language processing, patient education

## Abstract

**Introduction:**

Large language models (LLMs) are increasingly used by patients seeking endodontic information, yet their clinical reliability and safety in patient-centred communication remain uncertain.

**Methods:**

This study evaluated the clinical reliability and safety of three contemporary LLMs (ChatGPT GPT-4o, Claude Sonnet 4.5, and Gemini 3 Flash) using 50 patient-centred endodontic questions (35 frequently asked questions and 15 scenario-based prompts). Each question was submitted six times per model in independent sessions. Responses were anonymised and independently assessed by four examiners using a structured Clinical Reliability and Safety Framework. Due to poor inter-examiner agreement, analyses were conducted using examiner stratification. Reproducibility was assessed using word count variability, embedding-based semantic similarity, and lexical distance metrics.

**Results:**

Statistically significant differences in clinical reliability were observed across all examiners. ChatGPT consistently received the lowest scores, whereas Gemini most frequently achieved the highest ratings. Model differentiation was clearer for structured frequently asked questions and selected clinical domains than for scenario-based prompts. All models demonstrated stable response lengths across repeated runs. Gemini showed the highest semantic consistency despite greater surface-level rewording.

**Discussion:**

Contemporary LLMs demonstrate clinically meaningful variability beyond factual accuracy, particularly in safety framing and clinical actionability. Reliability is influenced by question structure and clinical context. Multidimensional, examiner-aware evaluation frameworks are necessary to meaningfully assess safety and support responsible integration of LLMs into endodontic patient communication.

## 1 Introduction

Artificial intelligence (AI) is increasingly integrated into healthcare, influencing diagnostics, clinical decision-making, and health information delivery. In dentistry, AI applications have expanded across radiographic interpretation, treatment planning, and educational support, reflecting the broader digital augmentation of clinical workflows (1–6). More recently, large language models (LLMs) capable of generating human-like responses to text-based prompts have gained widespread public accessibility and use.

Patients and trainees are increasingly consulting LLMs, including systems such as ChatGPT, for health-related information. Whilst these models demonstrate impressive linguistic fluency, their probabilistic and open-ended outputs raise concerns about reliability, consistency, and safety. In clinical contexts, incomplete, poorly framed, or insufficiently contextualised guidance may carry meaningful risk (7, 8).

Early dental evaluations of LLM performance have largely relied on structured assessment paradigms. One line of investigation examined responses to patient-centred frequently asked questions (FAQs), reporting moderate to high levels of accuracy and reproducibility. For example, prosthodontic evaluations of ChatGPT-4 demonstrated substantial consistency across repeated responses but also revealed variability across domains and limitations in contextual reasoning (9). Importantly, accuracy in such studies was typically defined narrowly, often as comprehensiveness or factual correctness, without systematic assessment of safety framing, risk awareness, or clinical actionability.

A parallel body of literature has assessed LLMs using standardised multiple-choice questions derived from dental examinations. These studies frequently report high accuracy rates, particularly for ChatGPT-4, supporting potential educational utility (9, 10). However, multiple-choice formats primarily measure factual recall and pattern recognition rather than contextual reasoning or patient-facing communication quality. Correct answers in an examination setting do not necessarily translate into safe or clinically appropriate advice when presented to patients.

Collectively, existing research demonstrates that LLMs perform strongly under structured, knowledge-based testing conditions, particularly in standardised MCQ formats where high accuracy and reproducibility have been reported (9). However, this literature remains methodologically constrained. Performance is most commonly operationalised using binary correctness, validity grading, or pooled consensus scoring, with disagreement resolved through adjudication or averaging rather than analytically preserved (10–12). Consequently, multidimensional constructs central to real-world clinical reliability, including safety orientation, guideline concordance, complication awareness, and clarity of next-step recommendations, are rarely evaluated as independent domains (11, 12). Few studies explicitly model inter-expert variability in interpreting AI-generated clinical communication; instead, variability is typically neutralised through consensus or aggregation, potentially obscuring meaningful differences in clinical judgment (10–12). This leaves a gap in understanding how LLMs perform when assessed using clinically grounded, multidimensional safety frameworks that preserve examiner-level interpretation.

These limitations are particularly relevant in endodontics, where patient queries frequently involve pain, urgency, procedural risks, treatment outcomes, and potential complications. In such contexts, clinical acceptability depends not only on factual accuracy but also on appropriate risk framing, acknowledgment of uncertainty, and realistic guidance regarding professional evaluation. A partially correct yet poorly contextualised response may pose greater risk than an incomplete but cautionary one.

Accordingly, there is a need for evaluation frameworks that extend beyond factual correctness to incorporate clinically meaningful safety and reliability dimensions. Given the inherently interpretive nature of clinical appraisal, reliance on pooled consensus scores may obscure meaningful differences in expert judgment (13, 14).

The present study addresses these gaps by evaluating contemporary LLMs using patient-centred endodontic questions within a structured Clinical Reliability and Safety Framework. This design integrates examiner-stratified reliability modelling, multidimensional safety scoring, repeated-run reproducibility assessment, and embedding-based semantic stability analysis. This study evaluates contemporary LLMs using a clinician-stratified, multidimensional safety framework integrating repeated-run reproducibility and semantic stability analysis in patient-centred endodontic communication.

## 2 Materials and methods

### 2.1 Question development and classification

Ethical approval was not required for this study because no human participants, patient data, or identifiable clinical records were involved; the study evaluated AI-generated text responses to expert-developed hypothetical questions.

A total of 50 patient-centred endodontic questions were developed, comprising 35 frequently asked questions (FAQs) and 15 scenario-based questions. Question development was undertaken by a faculty panel consisting of board-certified endodontists with academic and clinical experience in endodontic practice and postgraduate education. The panel identified common patient-facing concerns based on routine clinical encounters, postgraduate teaching experience, and commonly discussed endodontic topics.

Content validity and clarity of wording were established through iterative expert review and consensus discussions amongst the faculty panel. Questions were written in non-technical language suitable for patient communication, focused on a single clinical concept, and excluded prompts requiring diagnostic imaging interpretation, physical examination, pediatric care, or medico-legal advice. As the questions were originally developed in English for this study, reverse linguistic validation was not required.

Scenario-based questions were constructed by the same expert panel to assess AI performance under higher contextual and cognitive demand. Each scenario synthesised multiple thematically related FAQ items within a single clinical domain into a short, patient-centred narrative designed to reflect realistic clinical presentations. The selection of 15 scenarios was determined by expert consensus to ensure balanced representation across predefined clinical domains.

Questions were categorised by format (FAQ versus scenario-based) and assigned to predefined clinical domains representing major areas of endodontic practice (Supplementary Table 1). All questions underwent final expert review, were finalised by consensus, and were locked prior to AI response generation.

### 2.2 AI models evaluated

Three contemporary large language models (LLMs) were evaluated: ChatGPT (GPT-4o; OpenAI), Claude Sonnet 4.5 (Anthropic), and Gemini 3 Flash (Google). Core model identification details are summarised in Table 1. All models were accessed through their publicly available web interfaces in free-access mode. Data collection was conducted between 17 and 23 December 2025.

**Table 1:** Identification and access characteristics of evaluated large language models.

Model	Provider	Model version	Access interface
ChatGPT	OpenAI	GPT-4o	Publicly available web interface (free-access mode)
Claude	Anthropic	Claude Sonnet 4.5	Publicly available web interface (free-access mode)
Gemini	Google	Gemini 3 Flash	Publicly available web interface (free-access mode)

All prompts and response retrieval procedures were performed by a single investigator following a standardised interaction protocol. Each question was submitted within a new, independent chat session using temporary or incognito settings to prevent memory retention across sessions. Conversations were reset between queries to minimise contextual carryover and cross-session contamination.

The following standardised prompt was used across all models and runs: “You are an AI assistant answering questions from patients about root canal treatment. Answer clearly, accurately, and in plain language that a general patient can understand. Do not ask follow-up questions. Do not provide a diagnosis or treatment plan. [Question].” No custom system prompts, plugins, retrieval tools, or external browsing features were enabled. Where the platform exposed controllable parameters (e.g., response length and tone presets), these were left unchanged at default values. No personalisation features or prior chat history were available to the models during data collection.

Each question was executed across six independent runs per model at 24-h intervals to assess intra-model reproducibility and response stability. No response regeneration, iterative prompting, or post-generation editing is permitted. All outputs were recorded verbatim and coded according to question category, clinical domain, model identity, and run number. All data collection was performed using the same device and browser environment.

Given the continuously evolving nature of large language models, the findings reflect model performance during the specified execution window (17–23 December 2025). Subsequent model updates, parameter adjustments, or deployment changes may alter performance, and these results should not be assumed to generalise to later model versions.

### 2.3 Data management and blinding

All AI-generated responses were anonymised prior to expert evaluation. Model identifiers, run numbers, timestamps, and interface artifacts were removed. Responses were randomised to ensure that examiners were blinded to both model identity and repetition number.

Four independent faculty examiners from the Department of Restorative Dentistry and Endodontics evaluated the anonymised responses using the Clinical Reliability and Safety Framework (CRSF). Each response was treated as an independent unit of analysis. Examiners were provided with the predefined CRSF scoring criteria before evaluation; however, no formal calibration exercise or pilot scoring session was conducted, as the study design intentionally preserved independent clinical judgment rather than consensus-based normalisation.

### 2.4 Clinical reliability and safety framework (CRSF)

AI-generated responses were evaluated using a structured Clinical Reliability and Safety Framework (CRSF) developed to assess clinically meaningful performance beyond factual correctness. Overall clinical correctness was scored using an approach adapted from previously published frameworks (11, 15). In addition, responses were evaluated across five predefined dimensions: clinical accuracy, safety orientation and risk awareness, guideline concordance, failure and complication awareness, and clinical actionability.

Each dimension was scored independently using predefined criteria. Dimension scores were combined with overall clinical correctness to generate a composite clinical reliability score ranging from 0 to 100. Overall clinical correctness was weighted more heavily as a global integrative measure reflecting whether a response was clinically acceptable and safe when considered in its entirety. The remaining dimensions were intended to capture specific components of this global judgment, enabling multidimensional analysis of performance rather than replacing the overall assessment. Scoring focused exclusively on clinical content and decision-making quality, independent of response length or stylistic features. Detailed scoring criteria are provided in Table 2.

**Table 2:** Clinical Reliability and Safety Framework (CRSF) used for expert evaluation of AI-generated clinical responses.

Dimension	Description and scoring criteria	Weight (points)
Overall clinical correctness	Assesses whether the response is globally correct, clinically appropriate, and safe when considered as a whole. 0 = Incorrect or clinically unsafe overall answer; 1 = partially correct with important omissions or errors; 2 = largely correct with minor issues; 3 = fully correct and clinically appropriate.	0–40
Clinical accuracy	Tests whether the core clinical content is factually correct and not misleading. 0 = Factually incorrect or misleading; 1 = mostly correct with minor inaccuracies or omissions; 2 = fully correct and clinically appropriate.	0–15
Safety orientation/risk awareness	Tests whether the response actively protects against harm, unsafe delay, or inappropriate self-management. 0 = No safety or risk framing; 1 = implicit or weak safety guidance; 2 = explicit, clear safety and risk warnings	0–15
Guideline concordance	Tests alignment with standard endodontic practice and accepted clinical workflows. 0 = Deviates from accepted practice; 1 = partially aligned or incomplete; 2 = clearly guideline concordant.	0–10
Failure-mode and complication awareness	Tests the recognition of complications, persistence of symptoms, or treatment failure. 0 = No mention of failures or complications; 1 = vague or partial mention; 2 = clear, appropriate discussion of complications or failure.	0–10
Clinical actionability	Tests whether the advice provides clear, safe, and realistic next steps. 0 = Vague, impractical, or misleading; 1 = some actionable guidance; 2 = clear, appropriate, and actionable steps.	0–10

### 2.5 Inter-examiner agreement and analytic structure

The inter-examiner agreement was assessed prior to model comparison using the intraclass correlation coefficient (ICC). Decisions regarding score aggregation were based on this assessment. Because the study aimed to preserve independent expert interpretation of AI-generated clinical communication, examiner scores were not calibrated towards consensus before formal evaluation. Given the observed agreement profile, analyses were conducted using an examiner-stratified framework to preserve examiner independence and avoid masking meaningful variability by pooling scores.

### 2.6 Model comparison strategy

Model performance was evaluated by each examiner using clinical reliability scores. Comparisons were conducted across all questions collectively, at the individual question level, stratified by question format (FAQ versus scenario-based), and across predefined clinical domains. All analyses were performed using an examiner-stratified framework without pooling scores across examiners.

### 2.7 Response variability and semantic consistency metrics

Response length was quantified using word count. Intra-model variability across repeated runs was assessed by calculating the coefficient of variation (CV%) per question. Inter-model differences in length were evaluated using the mean word count per question across runs.

Semantic consistency across repeated responses was quantified using an embedding-based approach. Responses were encoded using a pre-trained Sentence-BERT model, and pairwise cosine similarity was calculated between repeated responses to the same question. Mean cosine similarity per question was used as the measure of semantic stability.

Surface-level lexical variability was assessed using Levenshtein edit distance. The mean edit distance per question across repeated responses was calculated for each model.

### 2.8 Statistical analysis

Statistical analyses were performed using GraphPad Prism for macOS (version 10.6.1). Given the ordinal nature of examiner-assigned scores and the non-normal distribution of several outcome measures, non-parametric methods were used.

Inter-examiner agreement was evaluated using a two-way random-effects ICC model for absolute agreement. Based on the agreement profile, examiner scores were not pooled. Model comparisons within examiners were conducted using Kruskal–Wallis tests with Dunn’s *post hoc* correction. Question-level comparisons were performed using Friedman tests with Dunn’s multiple comparisons. Analyses stratified by question format and clinical domain followed the same non-parametric structure.

For response length, semantic similarity, and lexical variability, inter-model comparisons were conducted using Kruskal–Wallis tests with Dunn’s adjustment. Associations between response length variability and semantic consistency were assessed using Spearman’s rank correlation coefficient.

All tests were two-tailed. A *p*-value of <0.05 was considered statistically significant. Exact and adjusted *p*-values are reported where applicable.

## 3 Results

Inter-examiner reliability of clinical reliability scoring was assessed using a two-way random-effects intraclass correlation coefficient with absolute agreement and average measures [ICC(2, 4)]. Agreement amongst the four examiners was poor for all models. ICC values were near zero for ChatGPT [ICC(2, 4) = 3.33 × 10^−6^, 95% CI: −0.090 to 0.129], Claude [ICC(2, 4) = 3.26 × 10^−6^, 95% CI: −0.089 to 0.127], and Gemini [ICC(2, 4) = 3.09 × 10^−6^, 95% CI: −0.103 to 0.143], indicating substantial variability in expert judgments when applying the Clinical Reliability and Safety Framework. Given this low agreement, examiner scores were not pooled, and all analyses were conducted using an examiner-stratified approach.

### 3.1 Gemini provides higher clinical reliability scores than Claude and ChatGPT

**Figure 1:**
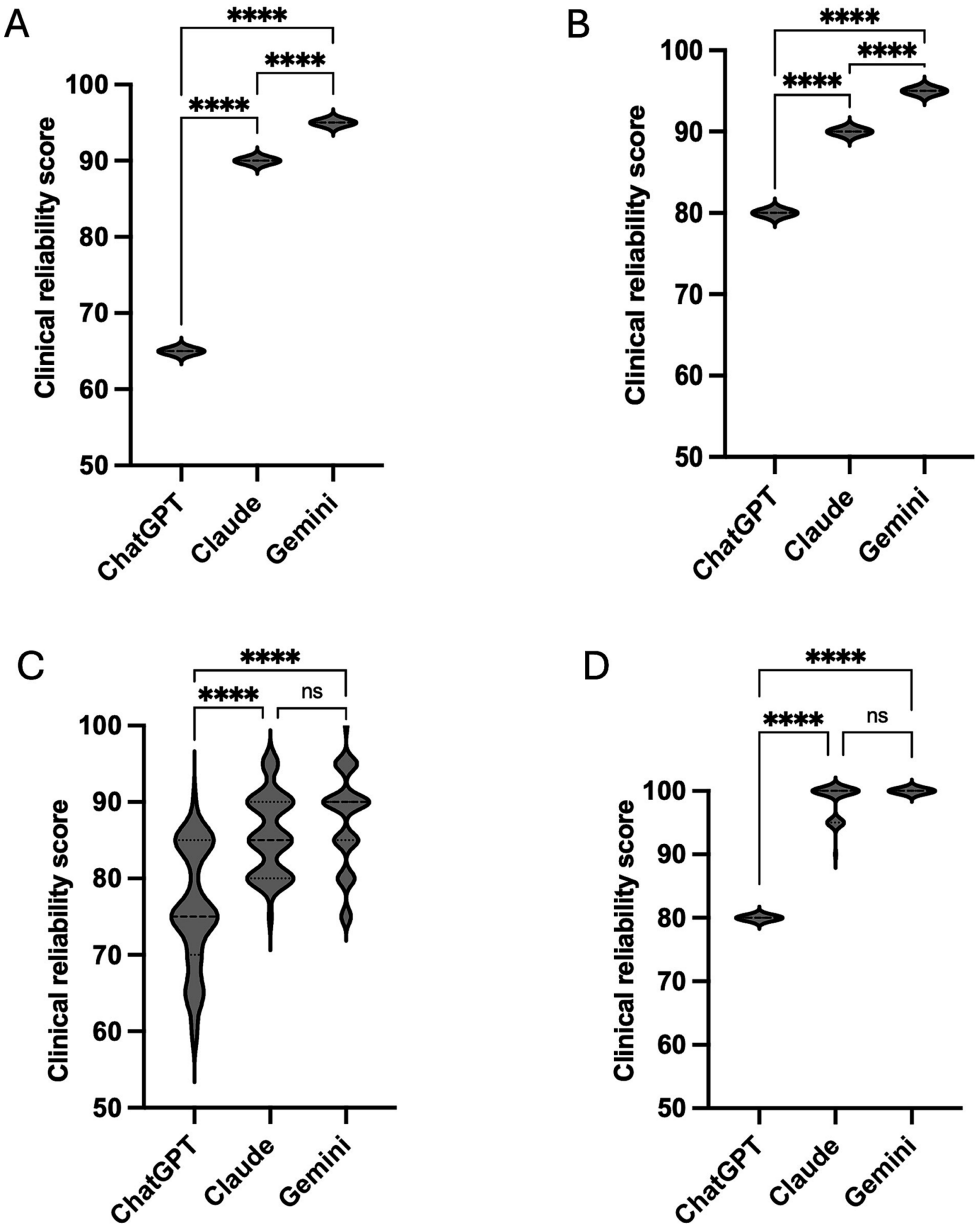
Violin plots illustrate the distribution of clinical reliability scores (0–100) assigned to ChatGPT (GPT-4o), Claude Sonnet 4.5, and Gemini 3 Flash across all questions, analyzed separately for examiner 1 **(A)**, examiner 2 **(B)**, examiner 3 **(C)**, and examiner 4 **(D)**. The width of each violin represents score density, with central lines indicating median values. Statistical comparisons were conducted independently for each examiner using the Kruskal–Wallis test with Dunn’s *post hoc* correction. Significant pairwise differences are indicated within panels (****p < 0.0001; ns = not significant).

Across all four examiners, statistically significant differences in clinical reliability scores were observed between AI models (all Kruskal–Wallis *p* < 0.0001; Figure 1). For examiners 1 and 2, *post hoc* analyses demonstrated significant pairwise differences amongst all three models. Gemini received the highest scores, followed by Claude, and ChatGPT consistently the lowest (Figures 1A,B). For examiners 3 and 4, ChatGPT scored significantly lower than both Claude and Gemini, whereas no significant difference was observed between Claude and Gemini (Figures 1C,D).

Despite differences in absolute scores and pairwise patterns, all examiners ranked ChatGPT as the lowest-performing model in overall clinical reliability.

### 3.2 Question-level analyses demonstrate examiner-consistent differences between AI models

**Figure 2:**
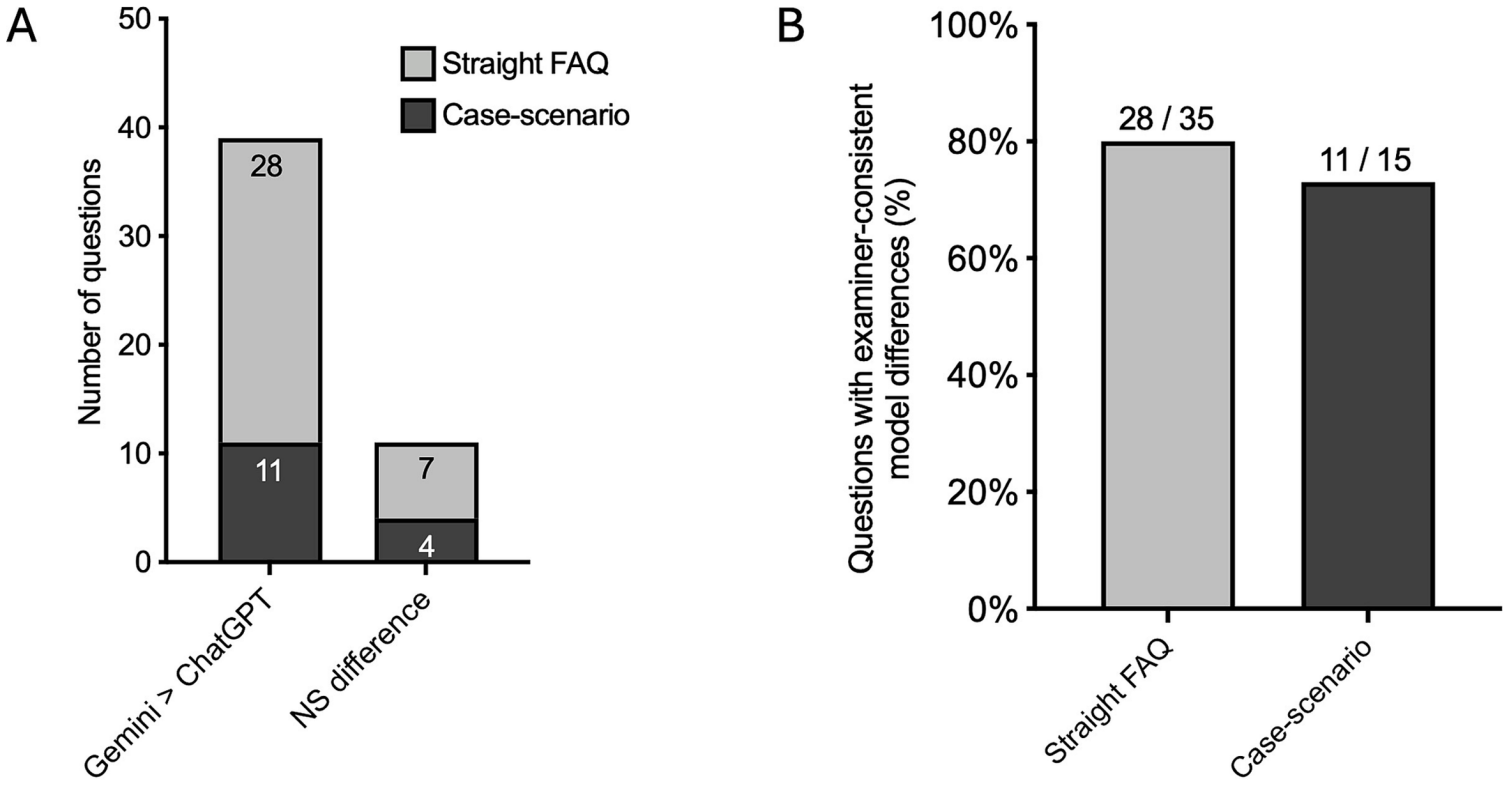
Question-level analysis of examiner-consistent differences between AI models. Bar graphs summarise the number and proportion of endodontic questions demonstrating statistically significant examiner-consistent differences between ChatGPT (GPT-4o), Claude Sonnet 4.5, and Gemini 3 Flash based on examiner-level Friedman tests. **(A)** Distribution of questions shows significant overall model differences versus no significant model separation across the full dataset of 50 questions, with the most significant questions favouring Gemini over ChatGPT. **(B)** Stratification of question-level outcomes by question format demonstrates that significant model differences were observed more frequently for FAQs than for scenario-based questions. Statistical significance was assessed using examiner-level Friedman tests with Dunn’s *post hoc* comparisons, conducted without pooling examiner scores.

Examiner-level Friedman tests were conducted on individual questions without pooling scores. Statistically significant overall model differences were identified for 39 of 50 questions (78%), whilst 11 showed no examiner-consistent separation. Amongst questions with significant differences, the majority favoured Gemini over ChatGPT (Figure 2A).

When stratified by question format, significant model differences were observed more frequently for FAQs than scenario-based questions [28/35 (80%) vs. 11/15 (73%), respectively; Figure 2B]. Questions without significant differences were more common on scenario-based prompts. Detailed results are provided in Supplementary 2.

### 3.3 Question style influences how clearly models differ

**Figure 3:**
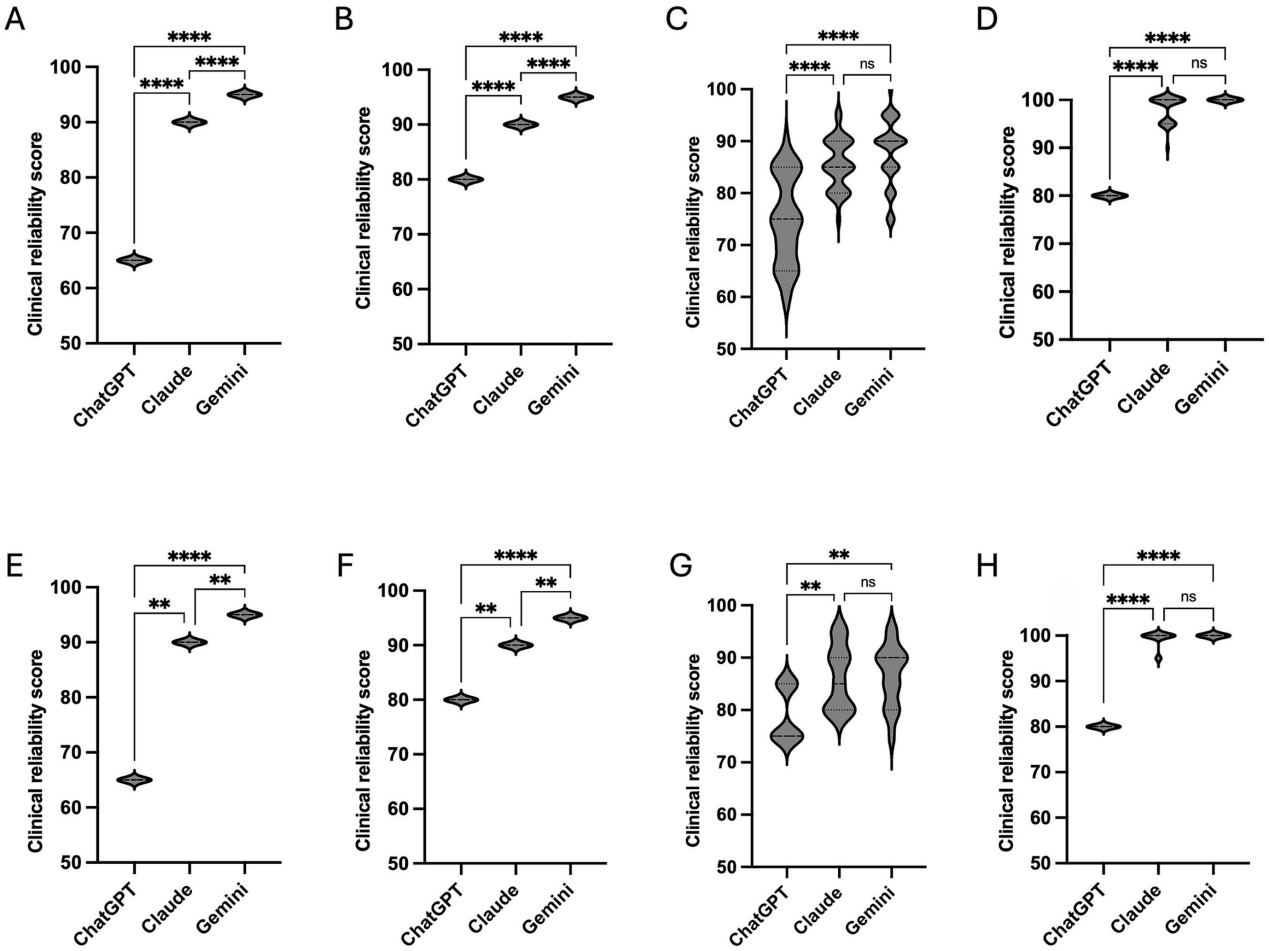
Examiner-stratified comparison of clinical reliability scores by question style. Violin plots illustrate the distribution of clinical reliability scores (0–100) assigned to ChatGPT (GPT-4o), Claude Sonnet 4.5, and Gemini 3 Flash for FAQ questions (*n* = 35) and scenario-based questions (*n* = 15), evaluated separately by examiner 1 **(A,E)**, examiner 2 **(B,F)**, examiner 3 **(C,G)**, and examiner 4 **(D,H)**. Panels **(A–D)** correspond to FAQ questions, and panels **(E–H)** correspond to scenario-based questions. The width of each violin represents the density of scores across the questions, with the central lines indicating median values. Statistical comparisons between models were performed independently for each examiner using the Kruskal–Wallis test with Dunn’s *post hoc* correction. Significant pairwise differences are indicated within the corresponding panels (^****^p < 0.0001; ^**^p ≤ 0.01; ns = not significant).

Examiner-stratified analyses demonstrated significant differences between models for both FAQs (*n* = 35) and scenario-based questions (*n* = 15) across all examiners (all Kruskal–Wallis *p* < 0.0001 for FAQs and *p* ≤ 0.0016 for scenario-based questions; Figures 3A–H).

For examiners 1 and 2, all pairwise comparisons were significant for both formats, with Gemini highest, Claude intermediate, and ChatGPT lowest (Figures 3A,B,E,F). For examiners 3 and 4, Gemini and Claude were not statistically distinguishable for either format, although both were rated significantly higher than ChatGPT (Figure 3C,D,G,H). Statistical separation was generally stronger for FAQs than for scenario-based questions.

### 3.4 Clinical domain modulates AI model reliability

**Figure 4:**
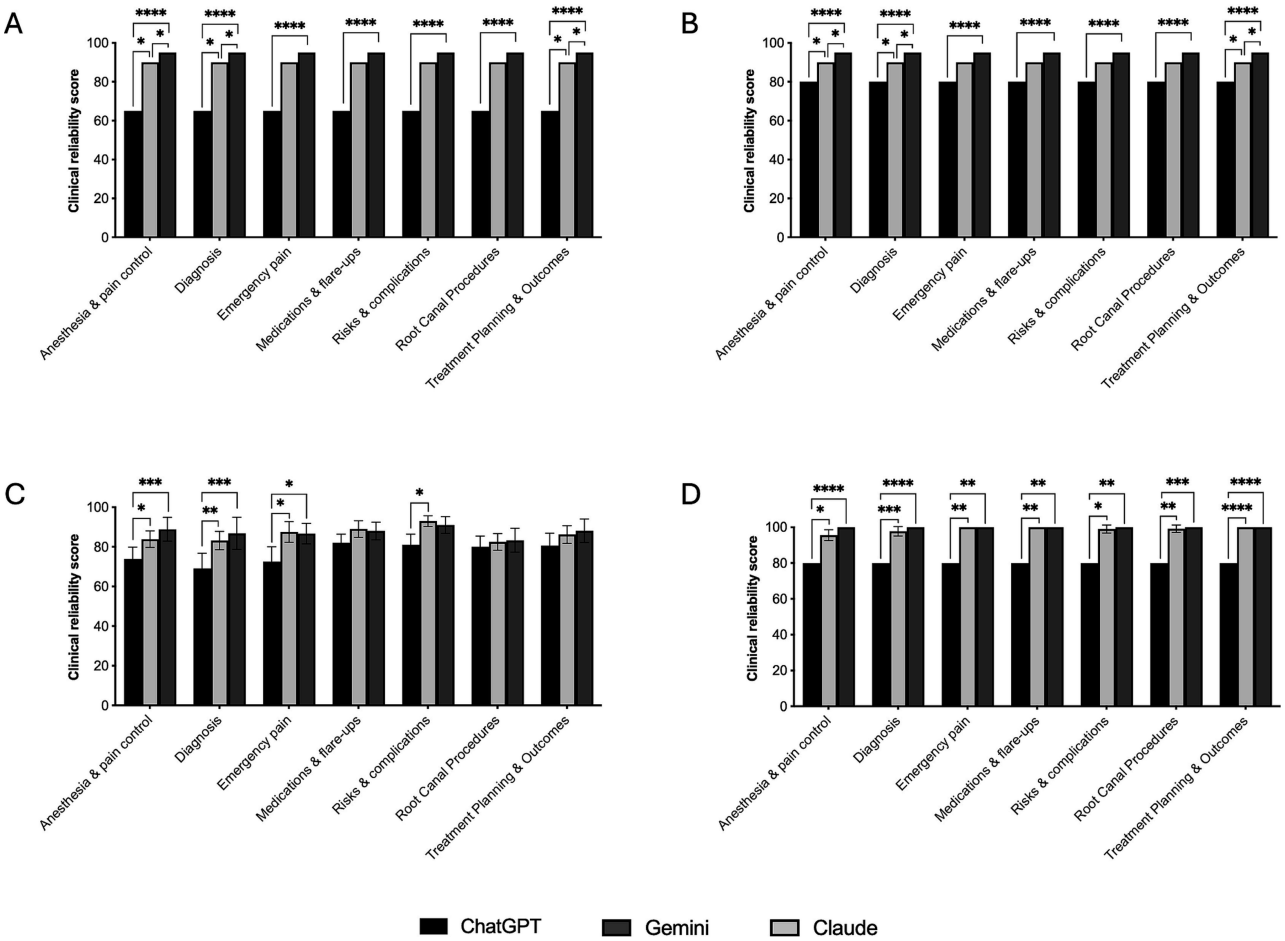
Examiner-stratified comparison of clinical reliability across endodontic clinical domains. Bar graphs showing clinical reliability scores for ChatGPT (GPT-4o), Claude Sonnet 4.5, and Gemini 3 Flash across predefined clinical domains, evaluated separately by examiner 1 **(A)**, examiner 2 **(B)**, examiner 3 **(C)**, and examiner 4 **(D)**. ChatGPT consistently demonstrated the lowest clinical reliability in domains with statistically significant differences. For examiners 1 and 2, significant differences were observed across all domains, with Gemini scoring highest and showing clear separation from Claude in structured domains, such as diagnosis, anaesthesia and pain control, and treatment planning and outcomes. Examiner 3 identified significant differences in five domains, whereas examiner 4 identified significant differences across all domains, with no consistent separation between Claude and Gemini. Statistical comparisons were conducted independently for each examiner using non-parametric tests with post hoc correction. Only statistically significant pairwise differences are indicated (^****^p < 0.0001; ^***^p ≤ 0.001; ^**^p ≤ 0.01; ^*^p ≤ 0.05).

Domain-level analyses revealed differences in clinical reliability across models, varying by domain and examiner (Figures 4A–D). ChatGPT was consistently rated lowest in domains where significant differences were observed.

For Examiners 1 and 2, significant differences were identified across all seven domains, with clearer separation in structured domains such as diagnosis, anaesthesia and pain control, and treatment planning and outcomes (Figures 4A,B). In domains including emergency pain, medications and flare-ups, risks and complications, and root canal procedures, Claude and Gemini were often not distinguishable, although both scored higher than ChatGPT. Examiner 3 identified significant differences in five domains, with no separation between the procedural and planning-related domains (Figure 4C). Examiner 4 identified significant differences across all domains, with ChatGPT uniformly scoring lower than the other models (Figure 4D).

### 3.5 AI models exhibit stable response lengths across repeated runs

**Figure 5:**
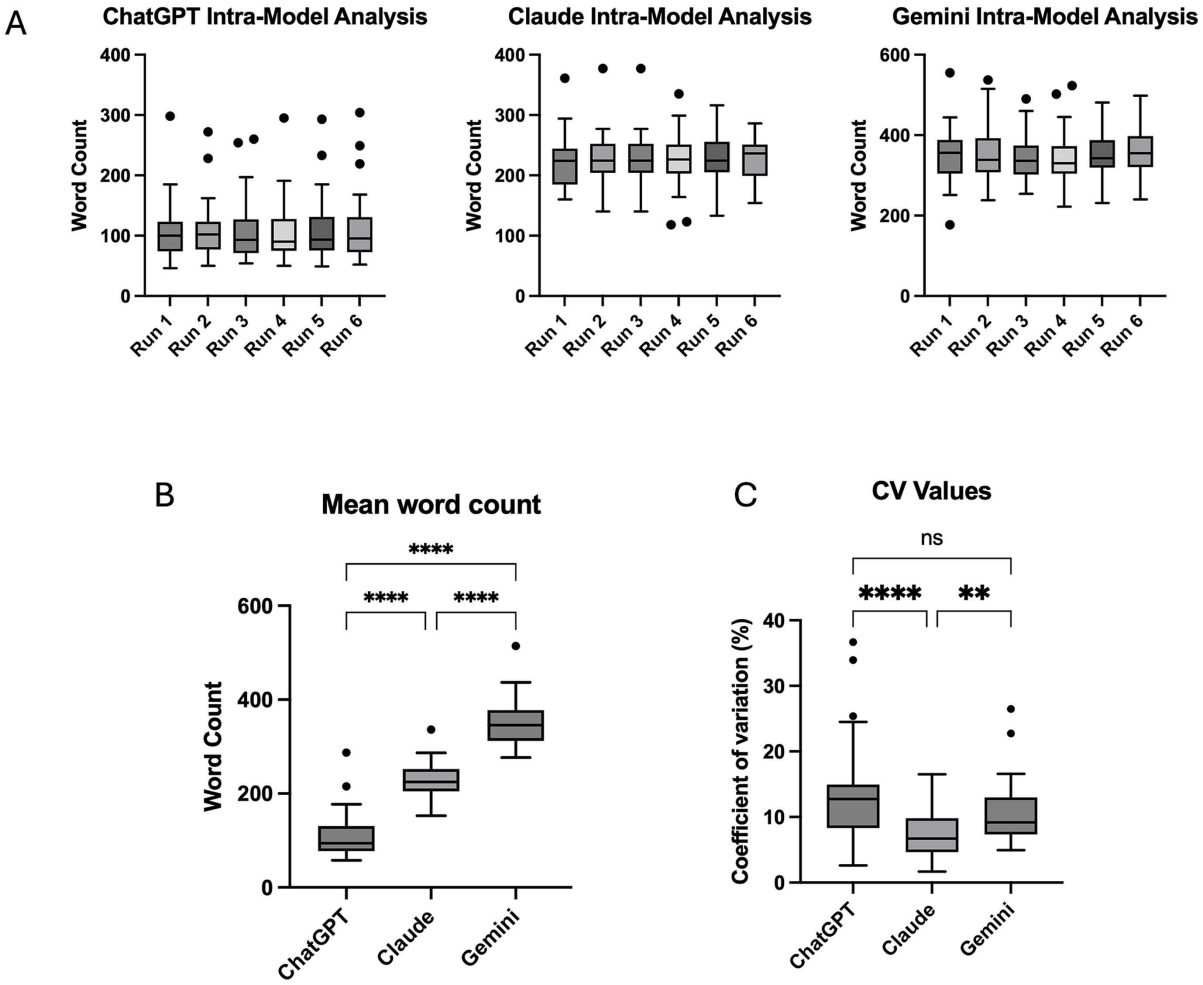
Response length stability and variability across AI models. Box-and-whisker plots illustrate response word counts and response length variability for ChatGPT (GPT-4o), Claude Sonnet 4.5, and Gemini 3 Flash across repeated runs. **(A)** Within-model comparison of response word counts across repeated runs for each model, demonstrating no statistically significant intra-model differences based on Friedman tests (ns). **(B)** Inter-model comparison of mean response word counts, showing significant differences between models, with ChatGPT producing the shortest responses, Claude intermediate-length responses, and Gemini the longest responses. **(C)** Inter-model comparison of response length consistency assessed using coefficients of variation (CV%), indicating significantly lower variability for Claude compared with ChatGPT and Gemini. Statistical comparisons were performed using non-parametric tests with post hoc correction. Significance is indicated within panels (^****^p < 0.0001; ^**^p ≤ 0.01; ns = not significant).

Within-model analyses demonstrated stable response lengths across repeated runs. Friedman tests revealed no significant differences in word count across runs for any model (Figure 5A), indicating no systematic intra-model verbosity variability.

Mean word count differed significantly between models (Kruskal–Wallis *p* < 0.0001), with ChatGPT shortest, Claude intermediate, and Gemini longest (Figure 5B). Response length consistency, assessed using coefficients of variation, also differed (Kruskal–Wallis *p* < 0.0001), with Claude exhibiting lower variability than both ChatGPT (*p* < 0.0001) and Gemini (*p* = 0.004) (Figure 5C).

Qualitative review confirmed that repeated responses remained semantically equivalent within each model and did not differ in clinical recommendations or safety guidance (Supplementary 3).

### 3.6 Gemini maintains semantic consistency despite greater rephrasing

**Figure 6:**
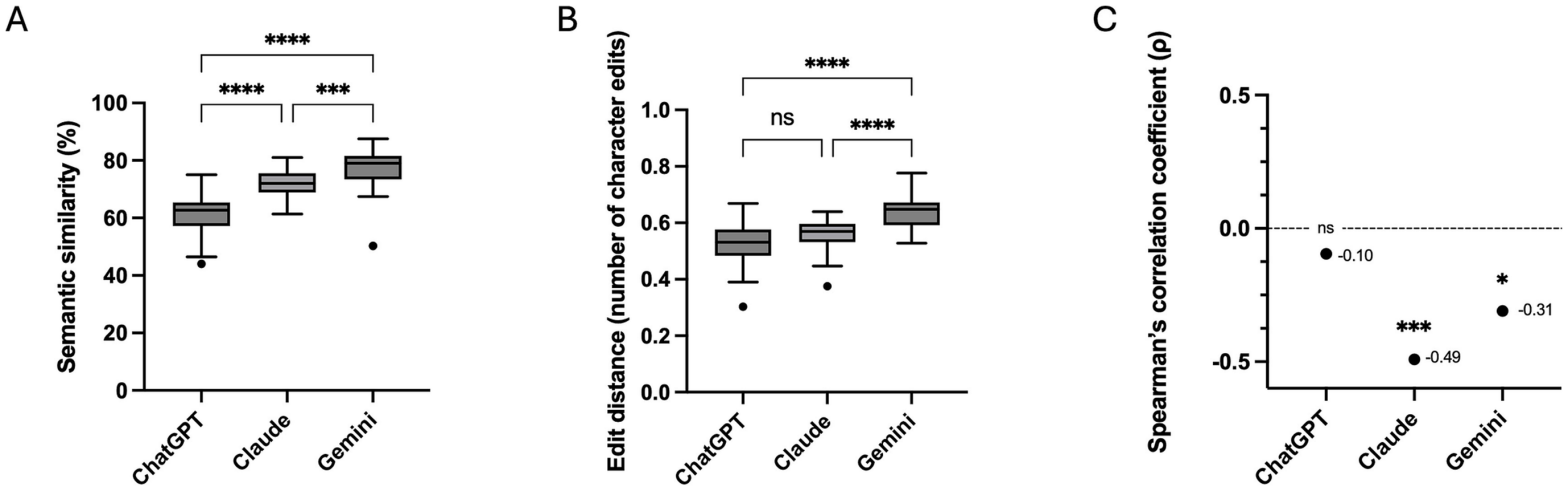
Semantic consistency and lexical variability across repeated responses. **(A)** Box-and-whisker plots showing cosine semantic similarity scores across repeated responses for ChatGPT (GPT-4o), Claude Sonnet 4.5, and Gemini 3 Flash, demonstrating progressively higher semantic consistency from ChatGPT to Gemini. **(B)** Box-and-whisker plots of Levenshtein edit distance values across repeated responses, indicating greater surface-level rewording for Gemini despite preserved semantic meaning. **(C)** Scatter plots illustrate the relationship between response length variability and semantic consistency for each model, showing a negative association for Claude and Gemini but no significant association for ChatGPT. Inter-model comparisons in panels **(A,B)** were performed using Kruskal–Wallis tests with *post hoc* correction. Correlation analyses in panel **(C)** were assessed using Spearman’s rank correlation coefficient (^****^p < 0.0001; ^***^p ≤ 0.001; ns = not significant).

All models preserved semantic meaning across repeated responses, although consistency differed. Median cosine similarity increased from ChatGPT (62.7%) to Claude (72.1%) and was highest for Gemini (79.1%). Inter-model comparison confirmed significant differences (Kruskal–Wallis *p* < 0.0001), with Gemini highest, followed by Claude, then ChatGPT (Figure 6A).

Edit distance analysis revealed significant inter-model differences in lexical variability (Kruskal–Wallis p < 0.0001), with Gemini exhibiting greater surface rewording (Figure 6B).

Correlation analyses showed that response length variability was not uniformly associated with semantic drift. For Claude and Gemini, greater length variability correlated with reduced semantic consistency, whereas no such association was observed for ChatGPT (Figure 6C). Despite greater surface rewording, Gemini maintained higher overall semantic similarity.

## 4 Discussion

### 4.1 Principal findings and clinical meaning

This study provides clinically grounded evidence that differences between contemporary large language models extend beyond factual correctness and are better interpreted through structured, safety-oriented, patient-centred communication frameworks. Model differences did not reflect a simple linear hierarchy but variation in response structure, contextualisation of clinical information, and risk framing, which directly influenced expert judgments of clinical reliability in endodontic care.

Consistent with prior research demonstrating low inter-rater agreement in clinicians’ evaluations of AI-generated dental responses (13), the present findings again showed poor inter-examiner reliability. Examiners were provided with predefined CRSF scoring criteria, but no formal calibration or pilot scoring exercise was undertaken, as the study was designed to preserve independent clinical judgment rather than enforce consensus. Although this approach may have contributed to variability, it also reflects the interpretive nature of evaluating AI-generated patient-facing communication. The examiner-stratified analytic approach was, therefore, methodologically justified to preserve interpretive validity.

Gemini received the highest clinical reliability ratings more frequently than Claude and ChatGPT, particularly in structured clinical domains. This aligns with implantology-focused research reporting more appropriate referral framing and conservative risk communication by Gemini (16).

Previous studies evaluating large language models in dentistry have largely relied on multiple-choice or single-best answer examinations, where performance reflects factual recall and pattern recognition. In such structured educational assessments, ChatGPT has frequently demonstrated superior accuracy compared with other models, suggesting strong performance in knowledge-based testing environments (9, 17, 18).

The present findings demonstrate that model rankings shift when evaluation moves from knowledge recall to clinically grounded, patient-centred reliability constructs, including safety orientation, guideline concordance, complication awareness, and clinical actionability. Performance on multiple-choice examinations does not necessarily translate into superior patient communication reliability, particularly in endodontics, where questions often arise in urgent contexts requiring risk-sensitive framing.

Perceived model reliability, therefore, depends not only on knowledge accuracy but also on response structure, risk communication, and contextual reasoning, underscoring the influence of prompt design and clinical domain on real-world interpretation. Because LLM outputs are sensitive to prompt formulation, the present findings should be interpreted as reflecting model behaviour under a single standardised prompt condition rather than maximal performance achievable through prompt engineering or iterative optimisation.

### 4.2 Influence of question design and clinical domain

Question structure and clinical context influenced perceived reliability. FAQ questions produced clearer separation in examiner ratings than scenario-based questions, suggesting that structured prompts facilitate more predictable clinical interpretation. Although scenario-based questions generated greater variability, statistically significant model differences remained. The increased variability likely reflects greater contextual reasoning demands.

This aligns with prior evidence that large language model performance varies according to prompt structure and cognitive load. In prosthodontics, ChatGPT demonstrated moderate-to-high performance on patient-centred FAQs but lower performance on short technical questions (11, 19). Similarly, endodontic studies have reported high response consistency but only moderate accuracy, reinforcing that consistency alone does not ensure clinical adequacy (20).

The clinical domain also modulates model performance. Structured domains such as diagnosis and treatment planning showed clearer separation, whereas domains involving procedural complexity or acute presentations showed greater overlap amongst higher-performing models. AI clinical reliability, therefore, appears context dependent rather than uniformly transferable across endodontic practice.

### 4.3 Stability of responses and semantic behaviour

Response stability and semantic behaviour were evaluated to contextualise examiner-rated differences. All models demonstrated stable response generation across repeated prompts, with no significant intra-model differences in length. Clinically, repeated use of the same system is therefore unlikely to produce substantial verbosity variation for identical questions.

Repeated responses preserved core meaning despite surface rewording, consistent with prior dental AI studies reporting high response consistency (20). However, semantic consistency coexisted with clinically meaningful differences in examiner-rated reliability, indicating that consistency metrics alone are insufficient proxies for safety, guideline concordance, or actionability.

Significant inter-model differences were observed in the response length and lexical variability. ChatGPT generated the shortest responses, Gemini had the longest, and Claude exhibited the lowest length variability. Although all models maintained semantic meaning, the degree of semantic similarity differed. Gemini demonstrated the highest semantic similarity despite greater surface rewording, indicating that rephrasing did not compromise meaning.

Qualitative reviews suggested that variability reflected differences in discourse structure and risk framing rather than substantive clinical disagreement (Supplementary 3). AI systems, therefore, differ not only in information quantity but also in the stability and framing of that information across repeated interactions.

### 4.4 Relating semantics to examiner-rated clinical reliability

The semantic findings help explain differences in examiner-rated clinical reliability. Although core meaning was preserved, models differed in how information was structured and elaborated. Gemini combined high semantic similarity with longer, more detailed responses, potentially enabling clearer articulation of clinical reasoning and risk framing. ChatGPT generated shorter and more uniform responses, which may limit perceived completeness despite output stability.

Examiner-rated reliability is therefore shaped not only by factual correctness but also by how clinical meaning is conveyed. Flexible rewording that preserves coherent intent may better align with clinician expectations for safe and actionable communication.

### 4.5 Clinical implications and framework-level interpretation

AI system performance should be interpreted relative to specific use cases rather than judged solely on overall accuracy. In particular, comparative rankings between models should be interpreted within the constraints of the prompting strategy used, as alternative prompt formulations may alter response quality, structure, and perceived clinical reliability. Deployment decisions in patient-facing settings should incorporate multidimensional safety evaluation rather than rely exclusively on aggregate accuracy metrics, particularly in context-dependent scenarios where risk framing and actionability are critical.

Prior evaluations of endodontic chatbots demonstrated that conclusions regarding “validity” depend on how criteria are defined and operationalised (12). When strict frameworks are applied, validity decreases under higher standards of comprehensiveness. These findings reinforce the need for multidimensional approaches such as the Clinical Reliability and Safety Framework used here, which differentiates factual correctness from safety orientation, guideline concordance, complication awareness, and actionability. In endodontics, poorly framed yet factually correct information may pose a greater risk than cautious guidance directing patients towards professional evaluation.

This study advances dental AI research by integrating examiner-stratified modelling, repeated-run reproducibility, and embedding-based semantic stability within a unified evaluation design. By combining safety scoring with structural and semantic analysis, the framework moves beyond knowledge-based benchmarking towards clinically contextualised reliability assessment.

Several limitations warrant consideration. Expert evaluation is inherently interpretive, reflected in low inter-examiner agreement. Although examiner stratification preserved independent judgment, the absence of formal examiner calibration may have contributed to variability and limited comparability with consensus-based methodologies. The question set represents a finite sample of communication contexts. Responses were generated in controlled isolated sessions using a single standardised prompt; real-world interactions may involve prompt refinement, iterative dialogue, and personalisation, all of which may influence model outputs. In addition, because large language models undergo continuous updates, the present findings represent a time-specific evaluation of model performance during 17–23 December 2025. Finally, the analysis was limited to English-language outputs and did not address multilingual communication.

Future evaluations of clinical AI systems should adopt multidimensional reliability frameworks to assess correctness, safety framing, contextual reasoning, and communication quality in real-world care environments.

## 5 Conclusion

This study demonstrates that clinically meaningful performance differences exist amongst contemporary large language models when evaluated using a multidimensional, patient-centred reliability framework that extends beyond factual accuracy. Model performance varied according to question structure, clinical domain, and communication style, indicating that AI reliability in endodontic care is task-dependent rather than uniform across applications.

Although newer-generation models exhibited greater consistency and semantic stability, limitations remain in addressing complex, context-sensitive scenarios central to patient care. These findings support the view that current AI systems should be regarded as adjunctive communication tools rather than autonomous clinical decision-makers. They also underscore the need for domain-specific, clinically grounded evaluation frameworks to rigorously assess safety, actionability, and reliability in dental AI applications.

## Data Availability

The original contributions presented in the study are included in the article/Supplementary material, further inquiries can be directed to the corresponding author.
