# Supplementary material for: Examiner stratification reveals clinically relevant variability in large language model answers to endodontic patient questions
Source: Front Med (Lausanne). 2026 Apr 22;13:1819087. doi: 10.3389/fmed.2026.1819087 (PMC13143729; doi:10.3389/fmed.2026.1819087)
Supplement: Supplementary file 1 [file Table_1.DOCX]

Table 1 Endodontic question categories by format and clinical domain used in the study.

| **ID** | **FAQ Question** | **Domain** |
| --- | --- | --- |
| Q01 | Will I feel pain during or after a root canal treatment? | Emergency pain |
| Q02 | Will there be swelling after a root canal, and how long does it usually last? | Emergency pain |
| Q03 | What is usually recommended if pain or swelling occurs after a root canal treatment? | Emergency pain |
| Q04 | Can painkillers alone treat the cause of tooth pain, or do they only relieve symptoms? | Emergency pain |
| Q05 | Why does toothache often get worse at night or while sleeping? | Diagnosis |
| Q06 | Why does my tooth hurt when I drink cold or hot beverages? | Diagnosis |
| Q07 | Does severe tooth pain always mean that the tooth cannot be saved? | Diagnosis |
| Q08 | Is it necessary to perform dental tests to find out what is wrong with my tooth? | Diagnosis |
| Q09 | Will dental tests make my tooth pain worse? | Diagnosis |
| Q10 | Are dental X-rays safe during pregnancy? | Diagnosis |
| Q11 | Why does my dentist ask about my past dental experiences and symptoms? | Diagnosis |
| Q12 | Do I need antibiotics for a root canal treatment? | Medications & flare-ups |
| Q13 | Are there any side effects from taking antibiotics for a root canal treatment? | Medications & flare-ups |
| Q14 | Can a tooth infection spread to other parts of the body if left untreated? | Medications & flare-ups |
| Q15 | How many appointments are usually needed to complete a root canal treatment? | Root canal procedures |
| Q16 | How long does each root canal appointment usually take? | Root canal procedures |
| Q17 | Can a root canal treatment be completed in one visit, or does it usually require multiple visits? | Root canal procedures |
| Q18 | Will the injection for local anesthesia hurt? | Anesthesia and pain control |
| Q19 | How long does the numbness usually last after a root canal procedure? | Anesthesia and pain control |
| Q20 | Can I still feel pain during a root canal treatment even with anesthesia? | Anesthesia and pain control |
| Q21 | What happens if the anesthesia does not work properly during the procedure? | Anesthesia and pain control |
| Q22 | Is it normal for my lip, cheek, or tongue to feel swollen or numb after anesthesia? | Anesthesia and pain control |
| Q23 | Can I safely drive home after receiving local anesthesia for a root canal? | Anesthesia and pain control |
| Q24 | What should I do if I start to feel pain while the root canal procedure is ongoing? | Anesthesia and pain control |
| Q25 | How successful are root canal treatments in general? | Treatment planning & outcomes |
| Q26 | How often do root canal treatments fail? | Treatment planning & outcomes |
| Q27 | Does the condition of the tooth affect the success rate of a root canal treatment? | Treatment planning & outcomes |
| Q28 | How long can a tooth last after a successful root canal treatment? | Treatment planning & outcomes |
| Q29 | Can a tooth infection return after a root canal treatment, and what signs might suggest this? | Root canal procedures |
| Q30 | Is there a risk of the tooth cracking or breaking after a root canal treatment? | Risks & complications |
| Q31 | Can instruments break inside the root canal during treatment, and what happens if this occurs? | Risks & complications |
| Q32 | Is pain or numbness after a root canal treatment normal, or could it indicate a complication? | Risks & complications |
| Q33 | Is there a chance that the filling material used in a root canal could extend beyond the root and cause problems? | Risks & complications |
| Q34 | What treatment options are available besides root canal treatment? | Treatment planning & outcomes |
| Q35 | What happens if I decide not to have a root canal treatment? | Treatment planning & outcomes |
| **Final Scenario Based Question** | | |
| S01 | I’ve had a tooth that hurts when I drink cold water. The pain sometimes lingers for a while after the cold is gone, and it seems worse at night. I’m nervous about the dental tests. Are they really necessary, and could they make my pain worse?” | Diagnosis |
| S02 | My tooth pain is severe and comes in waves. I’m scared that this means the tooth can’t be saved. Why does the dentist ask so many questions about my symptoms and past dental experiences before deciding what to do? | Diagnosis |
| S03 | I’m pregnant and having strong tooth pain. The dentist mentioned X-rays and tests to find the cause, but I’m worried about safety during pregnancy. Are dental X-rays and testing safe, and are they necessary to figure out what’s wrong? | Diagnosis |
| S04 | My tooth hurts with cold and sometimes with hot drinks too. I’m worried the pain means it’s too late to save the tooth. Does this type of pain automatically mean I’ll lose the tooth, or can it still be treated? | Diagnosis |
| S05 | I had a root canal yesterday. Today I’m sore and my face feels slightly swollen. How long can swelling last after a root canal, and what’s usually recommended if pain or swelling happens afterward? | Emergency pain |
| S06 | I’ve been taking painkillers for tooth pain, and they help for a few hours, but the pain gets worse at night and wakes me up. Are painkillers treating the actual cause, or only covering symptoms? Why does toothache often feel worse at night? | Emergency pain |
| S07 | I’m anxious about the local anesthesia shot for a root canal. Does the injection usually hurt, how long does the numbness last, and is it safe to drive home afterward?” | Anesthesia & pain control |
| S08 | During my last dental visit, I still felt pain even after getting numb. I’m worried the same could happen during a root canal. Can someone still feel pain during a root canal even with anesthesia, and what happens if the anesthesia doesn’t work properly while the procedure is happening? | Anesthesia & pain control |
| S09 | I have a tooth infection and I thought antibiotics were always needed for a root canal. My friend said antibiotics can have side effects. Do I need antibiotics for a root canal treatment, and what side effects should I know about? | Medications & flare-ups |
| S10 | I’ve been delaying treatment because I’m busy, but my tooth has been painful and I’m worried it might be infected. Can a tooth infection spread to other parts of the body if left untreated? What can happen if I decide not to do the root canal? | Medications & flare-ups |
| S11 | I’m trying to plan time off work. How many appointments are usually needed for a root canal, how long does each appointment take, and can it sometimes be completed in one visit? | Root canal procedures |
| S12 | I want to know what to expect long-term. How successful are root canals in general, does the condition of the tooth change the success rate, and how long can a tooth last after a successful root canal? | Treatment planning & outcomes |
| S13 | I had a root canal a few years ago, and now I’m noticing sensitivity and occasional discomfort again. Can a tooth infection return after a root canal, what signs suggest that, and how often do root canals fail?” | Root canal procedures |
| S14 | I read online that instruments can break during a root canal and that filling material can sometimes go beyond the root tip. After treatment, I also had lingering numbness and some pain. Are these things possible, what happens if they occur, and how can I tell what’s normal versus a complication?” | Risks & complications |
| S15 | My dentist recommended a root canal, but I’m unsure and worried the tooth could crack afterward. What treatment options are available besides a root canal, and what could happen if I decide not to have the root canal?” | Treatment planning & outcomes |
