# Supplementary material for: Examiner stratification reveals clinically relevant variability in large language model answers to endodontic patient questions
Source: Front Med (Lausanne). 2026 Apr 22;13:1819087. doi: 10.3389/fmed.2026.1819087 (PMC13143729; doi:10.3389/fmed.2026.1819087)
Supplement: Supplementary file 3 [file Table_3.DOCX]

**Supplementary 3: Qualitative Semantic Equivalence and Discourse Analysis of AI Model Responses**

This supplementary analysis provides a qualitative assessment of semantic equivalence and discourse-level variation in AI-generated responses across repeated runs and across models. The purpose of this appendix is to contextualize the quantitative semantic analyses by examining how differences in wording, structure, and tone relate to underlying meaning and clinical guidance.

**Analytic Approach**

For each of the 50 clinical questions, six independent responses were generated per model (ChatGPT, Claude, and Gemini) under identical prompting conditions. These responses were reviewed as parallel texts addressing the same communicative task. The analysis focused on whether repeated responses conveyed equivalent clinical meaning, whether any clinically meaningful differences emerged across runs, and whether systematic discourse patterns could be identified at the model level.

Semantic equivalence was assessed qualitatively by determining whether differences in phrasing, elaboration, or emphasis altered the interpretation of clinical advice, recommendations, or safety guidance. Differences were considered *not clinically meaningful* if the underlying clinical message remained unchanged. For each QuestionID–model pair, a single representative response was selected to illustrate the dominant and shared clinical content across runs.

**Semantic Stability Across Repeated Runs**

Across all models and questions, repeated responses consistently converged on the same underlying clinical meaning. Although responses varied in wording, sentence structure, level of detail, and rhetorical framing, no model produced conflicting advice, altered treatment recommendations, or inconsistent safety guidance when the same question was asked multiple times. In practical terms, stochastic variation affected *how* information was expressed rather than *what* information was conveyed.

This qualitative finding supports the quantitative semantic similarity results reported in the main manuscript and confirms that repeated prompting does not introduce clinically meaningful semantic drift within a given model.

**Surface-Level Variation Versus Meaning Preservation**

Qualitative inspection demonstrated that substantial surface-level variation, including differences in verbosity and rephrasing, often co-occurred with high semantic equivalence. Responses with higher word count variability or greater lexical rewriting still conveyed the same clinical propositions, timelines, and recommendations. Conversely, questions with lower semantic similarity scores tended to involve greater interpretive nuance or broader clinical context, warranting closer scrutiny but still rarely resulting in altered clinical guidance.

These observations reinforce the distinction between linguistic variation and semantic change and support evaluating AI outputs at the level of meaning rather than exact wording.

**Model-Level Discourse Tendencies**

Although semantic content was stable within and across models, recurring discourse patterns were observed:

- **ChatGPT** frequently adopted an explanatory or procedural register, emphasizing how treatments work and what patients should expect in general terms.
- **Claude** tended to produce concise, structured responses with clear conditional reasoning, symptom thresholds, and explicit guidance on when to seek professional care.
- **Gemini** consistently employed a reassurance-oriented and patient-centered tone, often normalizing symptoms before providing clinical explanations and management advice.

Importantly, these differences reflect stylistic and pragmatic variation rather than differences in clinical meaning or safety-relevant content. Representative responses illustrating these patterns are provided in Table S1 (example shown for the question, *“Will there be swelling after a root canal, and how long does it usually last?”*).

Table S1 Representative AI-generated responses illustrating qualitative semantic equivalence across repeated runs and models for an example clinical question.

| Will there be swelling after a root canal, and how long does it usually last? | | |
| --- | --- | --- |
| ChatGPT | Claude | Gemini |
| Yes, some swelling can happen after a root canal, but it’s not very common. If it does occur, it’s usually mild and part of the body’s natural healing process. The swelling may last for a few days—typically 2 to 3 days—and should gradually go down.  If swelling gets worse after a few days, is severe, or comes with a fever or strong pain, it could be a sign of infection, and you should contact your dentist. | Yes, some swelling after a root canal is normal and relatively common. Here's what you can typically expect:  How much swelling: Most people experience mild to moderate swelling in the area around the treated tooth. This might affect your gum, cheek, or jaw on that side of your face.  When it appears: Swelling usually peaks within 2-3 days after the procedure.  How long it lasts: For most patients, swelling begins to go down after those first few days and typically resolves completely within 3-7 days. The exact timeline varies from person to person.  What helps:   - Applying an ice pack to the outside of your face (20 minutes on, 20 minutes off) during the first 24-48 hours can reduce swelling - Taking any anti-inflammatory medication your dentist prescribed or recommended - Keeping your head elevated, even while sleeping - Getting adequate rest   When to contact your dentist: While some swelling is normal, you should reach out to your dentist if the swelling gets significantly worse after the first few days, is accompanied by fever, or if you develop severe pain or difficulty breathing or swallowing.  Everyone heals at their own pace, so some variation in swelling and recovery time is completely normal." | "It is common for patients to experience some level of swelling after a root canal. This is usually part of the body’s natural healing process in response to the procedure.  What to Expect with Swelling  Normal Swelling: You may notice mild puffiness or tenderness in the gums surrounding the treated tooth or even slight swelling in the cheek.  Peak Time: Swelling typically reaches its peak within 24 to 48 hours after the treatment.  Duration: In most cases, mild swelling begins to subside after the second or third day and should be significantly better within 3 to 7 days.  How to Manage It: Cold Compresses: Applying an ice pack to the outside of your face (20 minutes on, 20 minutes off) during the first 24 hours can help minimize inflammation.  Rest and Elevation: Keeping your head slightly elevated while resting can help reduce fluid buildup in the facial tissues.  Soft Foods: Sticking to a soft diet and chewing on the opposite side of your mouth can prevent further irritation to the area.  When to Contact Your Dentist: While minor puffiness is expected, you should reach out to your dental office if you experience any of the following ""red flag"" symptoms:   - Swelling that worsen safter the first three days rather than getting better. - Swelling that spreads toward your neck, eye, or under your jaw. - Severe pain that is not relieved by over-the-counter medication. - A fever, a persistent bad taste, or visible pus around the tooth.   Would you like me to provide tips on what foods are easiest to eat during root canal recovery?" |

**Summary**

Overall, this qualitative analysis demonstrates that AI-generated responses are semantically robust across repeated runs, with variation largely confined to surface linguistic realization and discourse strategy. Differences between models are best characterized as differences in tone, structure, and communicative emphasis rather than differences in clinical advice. This appendix supports the interpretation that observed semantic stability in the quantitative analyses reflects true preservation of clinical meaning rather than artifactually similar wording.
